# The gluteus maximus V-Y advancement flap for reconstruction of extensive soft tissue loss following an advanced sacral pressure ulcer. A case report and mini review

**DOI:** 10.1016/j.ijscr.2020.06.060

**Published:** 2020-06-17

**Authors:** Landry W. Tchuenkam, Flobert Titcheu, Aimé Mbonda, Trevor Kamto, Axel M. Nwaha, Igor J. Kamla, Joel Noutakdie Tochie

**Affiliations:** a Faculty of Medicine and Biomedical Sciences, University of Yaoundé I, Cameroon; b Njombe Saint Jean de Malte Hospital, Cameroon; c Urology Unit, Department of Surgery, Douala Laquintinie Hospital, Cameroon

**Keywords:** Pus, pressure ulcers, PIs, pressure injuries, WHO, World Health Organization, mmHg, millimeter of mercury, bpm, beats per minute, cpm, cycle per minute, cm, centimeter, l, liter, g, gram, mg, milligram, NPUAP, National Pressure Ulcer Advisory Panel, m^2^, square meter, mm^3^, Cubic millimeter, Pressure ulcer, V-Y advancement flap, sacrum

## Abstract

**Background:**

The occurrence of pressure injuries (**PIs**) in an inpatient is a serious medical condition that requires a rigorous clinical evaluation. Management of these lesions should be comprehensive, including general measures and local care. Wound care occupies a large part of the treatment. For large skin loss, the treatment requires plastic surgery techniques for reconstruction. Myocutaneous advancement flaps are a good therapeutic option due to the provision of vascularized tissue within the skin defect. To ensure the success of the surgery, a good surgical technique preceeded by careful preparation of the patient in conjunction with the anesthesia team is imperative.

**Case presentation:**

We report the case of a 60-year-old bed ridden man for more than two months following an ischemic stroke with left spastic hemiplegia and loss of sensitivity. He was transferred to our surgical unit for the management of a chronic stage 4 **PIs** in the sacral area complicated with cellulitis, subcutaneous abscess and severe sepsis. Initial management consisted of resuscitation measures combining fluid therapy, antibiotic therapy, analgesics, blood transfusion, nutritional support, physioteherapy, wound care as well as preventive measures and psychological support. Once the granulation tissues were fully developed, the patient underwent reconstruction surgery with a V–Y myocutaneous advancement flap. The graft was successful with complete wound healing thereafter.

**Conclusion:**

**PIs** are a multifactorial, complex and disabling pathology that requires multidisciplinary care. Surgeons, anesthetists, nutritionists, physiotherapists, nurses must act in agreement in order to provide optimal treatment. Prevention is the rule.

## Highlights

• Management of the pressure injuries (**PIs**) consisted of sequentially performed actions: wound debridement, cleansing, dressing and wound coverage.
• Wound coverage should be done once the local infection is treated, the debridement is done and the budding is obtained.
• Several processes can be performed for wound coverage: second intention healing, direct wound closure, skin grafting and fasciocutaneous flap or myocutaneous flap.
• The main operative steps concerning V–Y flap surgery are: flap drawing, flap edge incision, dissection follow by the lifting and transposition of the flap on the recipient area, completed by fixation of the flap in the recipient area and closure of the donor area under suction drains.

## 1 Background

### 1.1 Epidemiology & impact of PIs

Pressure Injuries (**PIs**) are a current contributor to morbidity, prolonged hospital stay and poor quality of life in the daily clinical practice. Hence, **PIs** are a major concern for public health [1,2] primary affecting the bed ridden patients, their family relatives responsible of paying their cost of healthcare in countries with no national health insurance such as most low-and middle-income countries. In Spain, the total cost of managing the pathology represents 5% of the annual expenditure of the health system in 2007 [3]. This cost varies depending on the stage of the **PIs** and associated complications [1,4]. The pathology remains frequent in hospital settings despite the progress made in the development of their risk assessment, treatment and prevention in terms of medical devices related to reducing pressure and improving patient’s rehabilitation. The occurrence of **PIs** generates an additional cost in the care of the patient, both financially and in terms of human resources (health staff and family of the patient) [1]. The epidemiology of this condition varies greatly depending on the clinical settings. Independently of the hospital settling, the incidence rate of hospital-acquired pressure injuries was estimated between 1.8–3.6% [1,5]. This incidence varies depending on whether the patients are evaluated on short or long-term bases especially in the intensive care unit where the highest incidences has been reported [5]. **PIs** can also occur in areas of the body in contact with the casts or other medical equipment or devices, where prevalence rates of 0.65% where observed [6].

### 1.2 Definition of condition

In 2016, the National Pressure Ulcer Advisory Panel (**NPUAP**) revised in the definition of "pressure ulcer" to "pressure injury" which better expresses the magnitude of the changes observed [7]. Indeed, initial minimal lesions may present as changes in skin pigmentation and later as ulcerations. The term "pressure ulcer" is therefore indicated when the integrity of the skin surface is compromised. PIs are defined as localized lesions of the skin and/or adjacent tissues of ischaemic origin occurring as a result of prolonged external compression to the skin [7].

### 1.3 Preference site and classification

Many areas of the body may be subject to the development of PIs. When the patient is in the supine position, the most frequent locations are: sacrum, iliac bumps and heel [1]. In lateral position, preferential sites are: iliac crest, greater trochanter of femur, medial and lateral malleolus and the lateral part of the knee. Several classification systems for PIs are described in the literature [7]. They are based on the degree of deep extension of the ischemic lesions. Currently, the most commonly used classification is from the National Pressure Ulcer Advisory Panel (NPUAP) [7]. This system identifies 4 increasing stages of severity, with subsequently two categories of specific lesions: Unstable and Suspected deep tissue pressure injury [7].

### 1.4 Treatment

It is crucial, aimed at wound healing, avoiding potentially fatal septic complications and improving the quality of life as well as rehabilitation. Management options advocated depend on the stage of the **PIs** and sequentially performed actions: wound debridement, cleansing, dressing and wound coverage. Debridement of the wound and systematic dressings alone can cure most of the wounds. In case of extensive soft tissue lost, several processes for reconstruction are described in the literature; ranging from graft to fasciocutaneous and myocutaneous flaps [8,9].

Herein, the authors report a case of a stage 4 sacral **PIs** whose definitive treatment consisted in a plastic surgery of the lost skin using a musculocutaneous flap of the gluteus maximus. An update on the management of this pathology was made thereafter. We discuss the literature by retrieving related articles through a Medline and Google search between December 2008 to December 2019 using the following keywords: pressure ulcer; injury, and reconstruction. This research has been reported in line with the SCARE criteria [10].

## 2 Case presentation

This is the case of a 60-year-old retired black African. He was transferred from the Department of Internal Medicine to the General Surgery Department of the Saint Jean de Malte Hospital in Njombe, Cameroon, for the treatment of a chronic wound in the sacral area. His past history was relevant only for poorly controlled hypertension with complications of a hemorrhagic stroke two month ago. His blood pressure was currently normal by using oral perindopril and indapamide. He is not diabetic and does not smoke cigarettes. His family and psychosocial histories were normal.

Physical examination on admission to our General Surgery Department revealed a fully conscious patient, temperature 38.9 °C, blood pressure 108/64 mmHg, heart rate 103 beats per minute, respiratory rate 22 breaths per minute, oxygen saturation 97% and a body mass index (BMI) of 17 kg/m^2^. His conjunctivae were pale. The patient had left spastic hemiplegia with symmetrical muscle strength at 1/5, as well as a decrease in pain sensitivity. The examination of skin showed a wound at the level of the sacrum which measured 12 × 6 cm, a depth of 3 cm exposing the bone. The bottom of the wound was necrosed with purulent exudation. The skin around the wound was inflammatory, reddish and sensitive. Generalized skin examination revealed multiple stage 1 and 2 pressure ulcers on the left plantar, left trochanteric, and around the left ear. Pyuria was also observed through a trans-urethral probe carried by the patient. The rest of the examination, in particular cardiac and pulmonary, was normal. Initial laboratory panel showed anemia at 7 g/dl (that was microcytic), leucocytosis at 14,000/mm^3^, serum creatinine slightly raised at 15 mg/l, and hypo-albuminemia at 28 g/l. Transaminases, blood glucose and blood ionogram were within normal range. Our working diagnosis was severe multifocal sepsis (urinary and cutaneous) complicated by severe anemia and associated with malnutrition and a stage one acute kidney injury. The sacral pressure ulcer was graded stage 4 with complications of cellulitis and a subcutaneous abscess.

Initial management consisted of resuscitation measures, including the placement of a large gauge, transfusion of 1500 mL of packed red cells and a 2000 mL vascular fluid load of normal saline over 24 h. He equally received broad spectrum antibiotic therapy using ceftriaxone 2 g/24 h intravenously (IV), metronidazole 500 mg/8 h IV; analgesics with paracetamol, tramadol and anticoagulant prevention with enoxaparin. The urinary catheter was removed and its tip underwent bacterial cultured which found a Proteus *mirabilis*. A broad-Specific antibiotic therapy was adapted accordingly. Thereafter the patient was equally placed on an anti-bedsore mattress; successive surgical debridements of necrotic tissues were done and samples sent for bacterial cultures and daily dressings. Nursing care was also aimed at ensuring a change of position of the patient every two hours with the help of the patient's family.

**Fig. 1:**
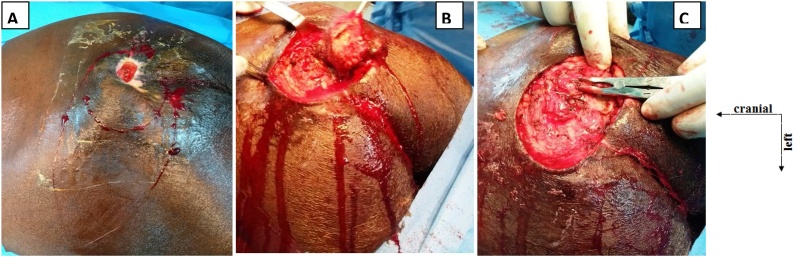
Intraoperative view of sacral pressure ulcer and excision of tissues. **A**: Aspect of ulcer and margins of excision. **B**: excision of ulcer margins and soft tissue debridement. **C**: bone curettage.
- After tracing the edges of the flap in V-shaped (Fig. 2)

After preoperative optimization of the patient, we continued daily wound dressing and preventive measures to ensure an adequate protein and carbohydrate caloric intake. Surveillance entailed daily inspection in search for the progression or healing of the **PIs** as well as looking for systemic signs of regression of sepsis such as fever, leukocytosis and sterile bacterial cultures. After 30 days of management the was no sign of sepsis, the wound was clean and had granulated remarkably but there was still considerable soft tissue lost; the second stage of management consisted of a reconstruction of sacral tissue lost in order to hasten wound healing. At this point his hemoglobin was 11.2 g/dl, well controlled hypertension (blood pressure 123/72 mmHg), He was fully conscious, had a motor power of 3/5 on both limbs of the side of hemiplegia, normal renal function test, BMI of 23 kg/m^2^, serum albuminaemia of 68 g/l and the wound granulation tissue were pinkish, a factor of successful wound graft. We opted for a myocutaneous flap of the gluteus maximus in V–Y under general anesthesia. The patient was placed in a prone position after intubation. The head and torso supported on a surgical frame and pillows. The main operative steps were:

- Wide debridement of the soft tissues (Fig. 1) and bone curettage.

**Fig. 2:**
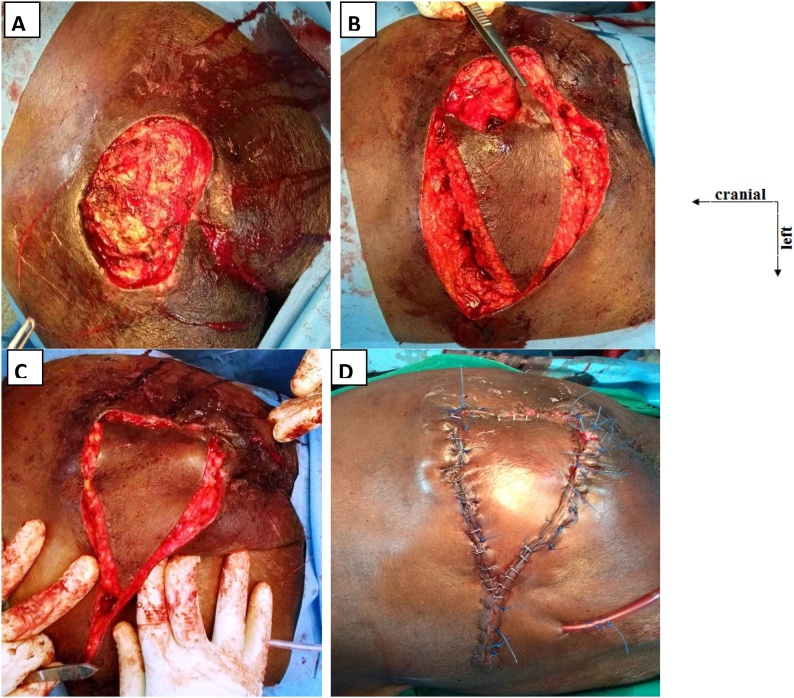
Intraoperative view of flap mobilization. **A**: drawing margins of the flap. **B**: incision of the margins of the flap and start of mobilization. **C**: end of flap mobilization. **D**: flap fixation, direct suture of the donor area under suction drain.
- we performed an incision of the flap margins with muscle dissection (Fig. 2**B**),
- followed by mobilization of the flap and advancement of the broad base of the V into the defect (Fig. 2C)
- After verification of haemostasis,
- we proceeded to suture the margins of the flap closed in layers and the defect was closed in a Y-shape (Fig. 2D)
- We left in place a drain for four days (Fig. 2D).

The postoperative outcome was marked by the occurrence of a little hematoma which was evacuated after the release of a few sutures. Postoperative care consisted of an intensification of pressure ulcer prevention measures associated with wound care, analgesics, antibiotics and physiotherapy. Complete scarring of the wound was obtained at one month post surgery.

## 3 Discussion

### 3.1 Pathogenesis and risk factors

The pathogenesis of the disease is complex. Tissue lesions occur when external forces extend over the soft tissues facing the bony prominences or under a medical or other device. These external forces act alone or in combination on the soft tissues. This is the pressure exerted by the support, friction, shear forces and maceration [11]. These extrinsic factors alone cannot explain the occurrence of **PIs**. They interact with intrinsic factors, specific to the patient: immobility, malnutrition, reduced perfusion in skin area, sensory skin disorders, aging skin and psychiatric disorders of the patient [7,11]. Immobility is the main risk factor for the patient that conditions the occurrence of PIs [11].

### 3.2 Initial clinical assessment and related classification

PIs is a complex pathology whose imperative management must integrate a complete, loco regional and general clinical evaluation [2,12]. The assessment of the patient's general medical condition consists of identifying comorbidities, exogenous and endogenous risk factors present in the patient. Loco regional examination should focus on areas of prominent bone. All skin lesions should be characterized in terms of: site, measurements (length, width, and depth), wounds edges, presence of necrotic tissue, purulent exudates, wound odor and the condition of the wound periphery [12]. Signs of good granulation should also be noted. This local assessment should result in the classification of lesions according to the stages of NPUAP [7]. This classification determines local care, prognosis, and length of hospital stay (Table 1).

**Table 1:** Clinical staging of pressure Injuries and epidemiology.

Stade*	Descritpion*	frequency¶	time to healing‡	Cost of wound care‡
**1**	Intact skin with a localized area of nonblanchable erythema after relief of pressure	27.9%	1.1	£1382.17
**2**	Blister or Partial-thickness loss of skin with exposed dermis	41.8%	5.0	£8663.34
**3**	Full-thickness skin loss, in which Subcutaneous fat may be visible. Fascia, muscle, tendon, ligament, cartilage, or bone is not exposed	4.5%	7.7	£9714.47
**4**	Full-thickness skin and tissue loss with exposed fascia, muscle, tendon, ligament, cartilage, or bone in the ulcer	1.8%	7.7	£10065.42
**Unstageable**	Full-thickness tissue loss in which the extent of tissue damage cannot be confirmed because the base of the ulcer is covered by slough and/ or eschar in the wound bed	10.3%	10.	£9085.49
**Suspected deep tissue injury**	Intact or non intact skin with localized area of Purple or maroon discoloration or blood-filled blister	12.1%	NR	NR

**NR**: not reported; **£**: pound sterling.

* Classification of lesions according to the stages of NPUAP [7].

¶ Distribution of frequency based on reference [5].

‡ Data from reference [4].

### 3.3 Complications

The second step of the clinical evaluation is to look for the complications of **PIs**. The most common are infectious complications [13], most often a polymicrobial infection. The clinical manifestations are diverse. The infection can be superficial. In this case we must look for signs of inflammation such as heat, erythema, local pain and the presence of a purulent exudates [11]. The infection can also be deep and manifest as cellulitis, subcutaneous abscess, pyomyositis, osteomyelitis or bacteremia with septicemia [12]. Generally, there is an underlining infection in any **PI** whose healing is delayed. In addition to infectious complications, there are complications related to **PIs** [13], namely sinus tract formation with neighboring organs, secondary systemic amyloidosis, malnutrition [14] and more rarely carcinoma in chronic ulcers. Muscle retractions and the psycho-social impact related to the pathology must also be evaluated and taken into account in the treatment

### 3.4 Management: general care for optimizing medical status

The general aims of management are to [2,12]: research and correct risk factors, intensify prevention measures, assess and treat pain, assess and manage local and distant infections. In addition, it is also important to ensure adequate fluid intake [14]; find and correct a nutritional deficit [14] and provide psychological support [12]. Local treatment varies depending on the stage of the **PIs**. It obeys the same principles as the management of all chronic wounds: debridement, cleansing of the wound and wound dressing [12,15]. The goal is to promote the granulation of the wound and the cutaneous coverage. Debridement involves the removal of necrotic or ischaemic tissues. It can be mechanical, enzymatic, chemical, using larvae and surgical [12]. For wound dressing, many antiseptics used in dressings were evaluated. It appears that none showed a superiority over the others [16]. The principle of care is to avoid desiccation of the wound or maceration. Indeed, the type of dressing should be adapted to the healing phase of the wound. Before a dressing is applied, wound cleansing of the ulcer using normal saline to aid removal of exudates and contaminants must be carried out regardless of the stage of the **PIs** [12].

### 3.5 Wound coverage

With regards to wound coverage, once the local infection is treated, the debridement is done and the budding is obtained, the next step of the treatment consists in ensuring the cutaneous cover. The latter consists of the addition of new tissue into the wound. Several processes have been described in the literature [8,9]. They vary according to the site of **PIs**, the depth, the cutaneous surface, the general state and mental status of the patient [9]. To achieve this goal, second intention healing, direct wound closure, skin grafting, fasciocutaneous flap or myocutaneous flap may be used [8,9]. Table 2 presents the various recovery methods, their advantages as well as their disadvantages. Reconstructive surgery involves deep ulcers which are large and difficult to heal on their own. It begins with the surgical preparation of the patient [17]. Indeed, few patients with PIs are eligible for surgery [9,18]. Nutritional status, including hypo albuminemia should be corrected before any surgery. The digestive and urinary preparation should determine the possibility of a temporary digestive and / or urinary diversion in order to reduce maceration and microbial proliferation at the operative site. Any muscular contracture must be treated beforehand, as well as a psychiatric disorder. This preparation will be done in concert with the team of anesthesiologists [17]. Once in the operating room, installation of the patient will depend on the site of **PIs**. The first step of the surgical procedure begins with the excision of devitalized, necrotic and suspicious tissues [12]. Excision must be carcinological; it may also interest parts of the bone affected. Once this debridement is done, the second step in the same operating time is the application of the flap. This begins with the flap drawing, then the flap edge incision, the dissection followed by the lifting and transposition of the flap on the recipient area [18]. The procedure is completed by attaching the flap in two planes to the recipient area and closing the donor area under suction drains [18]. At the level of the sacrum, several flaps have been described. We quote the myocutaneous flap of gluteus maximus in V–Y; the Griffith rotation flap and the Dufourmentel LLL flap [18]. Thiessen in 2008 showed that there was no significant difference in terms of early complications and recurrences between fasciocutaneous or myocutaneous flaps [19].

**Table 2:** Wound coverage options for pressure ulcer after debridement.

Reconstruction options	indications	comments
• **Option: Healing by secondary intention**		
	– Superficial stage 1 and stage 2 PUs – small size stage 3 PUs	– requires regular monitoring – requires regular dressings – Variable hospital – It can be assisted by the VAC*
• **Option: Direct wound closure**		
	– stage 3 Pus where the lack of tissue is not too great	– Rarely done, – Risk of suturing under stress that may cause skin necrosis and dehiscence of the wound – short hospital stays – Aesthetic scar
**• Option: Tissue expansion**		
	– stage 3 to stage 4 – small skin defect	– Consists of mechanical and gradual traction of the skin and subcutaneous tissue surrounding PUs, using a tissue expander or traction dressing
• **Option: Skin graft (split-thickness or full-thickness)**		
	– stade 3 or 4 PIs	– requires a well-granulating pressure ulcers – should not be applied on sites where pressure and stretching forces are important – Easy to pick up and apply – requires immobility of the patient to ensure the maintenance of the graft – *The lighter a graft is, the easier it takes, but the less aesthetic it is*
• **Option: Flaps**		
**Fasciocutaneous flaps**	– stage 3 or 4 Pus with large defect	– Axial flaps who carrying well-defined vessels have better outcomes
**Myocutaneous flaps**	– stage 3 or 4 Pus with large defect	– Method of choice for deep ulcers – provides good cushioning of the ulcer on the support areas, good vascularization of the flaps – the donor site usually closed directly
**Free flaps**	– stage 3 or 4 Pus with large defect	– Last alternative when all options have been exceeded – muscle-type flaps where the flap is removed from the donor site with its vascular pedicle and transplanted to the recipient site with vascular anastomosis by microsurgery

Reconstruction options and indications are based on the data from articles referenced in [13,14].

* VAC: vacuum-assisted closure.

The main complications of such procedures reside in the postoperative period; the surveillance will have to be rigorous, in search of the complications peculiar to the surgery of the flaps. They occur in about 58.7% [20]. The most frequent are: the disunion of the flap (dehiscence) (31.2%); recurrences 28.6%; infection (6.5%); hematomas, seromas and partial or total necrosis of the flap are less frequent [20].

### 3.6 Prevention

The optimal management of this pathology is through prevention. The related measures must be systematically applied to all bedridden patients [21]. The occurrence of PIs is a warning signal for the reinforcement of these measures which consist in [2,21]: identification of risk factors and evaluation of the risk of pressure sores by scales, use of pressure redistribution surfaces (static and dynamic supports), and an active mobilization of the patient at least every two hours to relieve tissue pressure. Other measures consist to maintain the skin hygiene, ensure adequate nutritional intake, promote education of the patient, the family and medical staff [21,22]. In clinical practice, bedsore risk assessment scales have been established. The scales of Norton and Braden are the most used in Anglo-Saxon countries [21]. These scales should be used in conjunction with clinical judgment for the identification of at-risk patients for whom armed surveillance and intensification of the previously mentioned preventive measures would be indicated [2].

## 4 Conclusion

Surgery in the context of **PIs** is part of a global policy for the management of these lesions. The reconstruction techniques are numerous and diverse. The nutritional, digestive, urinary, psychological and neurological preparation of the patients strongly influences the success of the surgery. It is a condition that affects the quality of life of the patient and those around him, increases the length of hospital stay, the overall cost of healthcare and contributes to premature death in hospitalized patients. As a result, each health facility should have a strict health policy in terms of prevention of these lesions.

## Declaration of Competing Interest

The authors do not declare any conflicts of interest.

## Funding

There is currently no source of funding for this research.

## Ethical approval

As a Case Report, the study is exempt from ethnical approval in my institution.

## Consent

Written informed consent was obtained from the patient for publication of this case report and any accompanying images.

A copy of the written consent is available for review by the Editor-in-Chief of this journal.

Any identifying material has been removed, including the patient's name, date of entry, face or any distinctive features on the pictures taken.

## Author contribution

Landry W Tchuenkam and Flobert Titcheu, contributed in the design of the study and writing of the manuscript.

Joel N Tochie, Axel Nwaha Makon, Igor J Kamla and Trevor Kamto, contributed in critical reading.

Landry W Tchuenkam, collected the pictures, and obtained the patient’s consent.

All authors have read and approved the final version of the manuscript.

## Registration of research studies

NA.

## Guarantor

Landry Wakheu Tchuenkam as corresponding author accept the full responsibility for this research.

## Provenance and peer review

Not commissioned, externally peer-reviewed.

## References

[1] Bauer K., Rock K., Nazzal M., Jones O., Qu W. (2016). Pressure ulcers in the United States’ inpatient population from 2008 to 2012: results of a retrospective nationwide study. Ostomy Wound Manage..

[2] Pressure ulcers | Guidance and guidelines | NICE [Internet]. [cited 2018 Dec 8]. Available from: https://www.nice.org.uk/guidance/qs89/chapter/about-this-quality-standard.

[3] Soldevilla Agreda J.J., Torra I., Bou J.E., Posnett J., Verdu Soriano J., San Miguel L., Mayan Santos M. (2007). The burden of pressure ulcers in Spain. Wounds Compend. Clin. Res. Pract..

[4] Guest J.F., Fuller G.W., Vowden P., Vowden K.R. (2018). Cohort study evaluating pressure ulcer management in clinical practice in the UK following initial presentation in the community: costs and outcomes. BMJ Open.

[5] Bergquist-Beringer S., Dong L., He J., Dunton N. (2013). Pressure ulcers and prevention among acute care hospitals in the United States. Jt. Comm. J. Qual. Patient Saf..

[6] Kayser S.A., VanGilder C.A., Ayello E.A., Lachenbruch C. (2018). Prevalence and analysis of medical device-related pressure injuries: results from the international pressure ulcer prevalence survey. Adv. Skin Wound Care.

[7] Edsberg L.E., Black J.M., Goldberg M., McNichol L., Moore L., Sieggreen M. (2016). Revised national pressure ulcer advisory panel pressure injury staging system: revised pressure injury staging system. J. Wound Ostomy Cont. Nurs..

[8] Wong J.K., Amin K., Dumville J.C., Cochrane Wounds Group (2016). Reconstructive surgery for treating pressure ulcers. Cochrane Database Syst Rev [Internet].

[9] Sørensen J.L., Jørgensen B., Gottrup F. (2004). Surgical treatment of pressure ulcers. Am. J. Surg..

[10] Agha R.A., Borrelli M.R., Farwana R., Koshy K., Fowler A.J., Orgill D.P. (2018). The SCARE 2018 statement: updating consensus surgical CAse REport (SCARE) guidelines. Int. J. Surg..

[11] Anders J., Heinemann A., Leffmann C., Leutenegger M., Pröfener F., von Renteln-Kruse W. (2010). Decubitus ulcers: pathophysiology and primary prevention. Dtsch Ärztebl Int..

[12] European Pressure Ulcer Advisory Panel, National Pressure Ulcer Advisory Panel (U.S.) (2014). Pan Pacific Pressure Injury Alliance. Prevention and Treatment of Pressure Ulcers: Quick Reference Guide.

[13] Moiziard A.-S., de Saint-Léger A.-S., Meaume S. (2009). pratique soignante - Les complications des escarres. http://www.em-consulte.com/en/article/206153.

[14] Saghaleini S.H., Dehghan K., Shadvar K., Sanaie S., Mahmoodpoor A., Ostadi Z. (2018). Pressure ulcer and nutrition. Indian J. Crit. Care Med. Peer-Rev..

[15] Qaseem A., Humphrey L.L., Forciea M.A., Starkey M., Denberg T.D., Clinical Guidelines Committee of the American College of Physicians (2015). Treatment of pressure ulcers: a clinical practice guideline from the American College of Physicians. Ann. Intern. Med..

[16] Reddy M., Gill S.S., Kalkar S.R., Wu W., Anderson P.J., Rochon P.A. (2008). Treatment of pressure ulcers: a systematic review. JAMA.

[17] (2016). Flap Coverage of Pressure Sores [Internet]. https://www.clinicalpainadvisor.com/anesthesiology/flap-coverage-of-pressure-sores/article/581917/.

[18] Rimareix F., Delpit X., Bauer T., Lortat-Jacob A. (2007). Traitement chirurgical des escarres. EMC - Tech Chir - Orthopédie - Traumatol..

[19] Thiessen F.E., Andrades P., Blondeel P.N., Hamdi M., Roche N., Stillaert F. (2011). Flap surgery for pressure sores: should the underlying muscle be transferred or not?. J. Plast. Reconstr. Aesthetic Surg..

[20] Bamba R., Madden J.J., Hoffman A.N., Kim J.S., Thayer W.P., Nanney L.B. (2017). Flap reconstruction for pressure ulcers: an outcomes analysis. Plast. Reconstr. Surg. Glob. Open.

[21] Qaseem A., Mir T.P., Starkey M., Denberg T.D., Clinical Guidelines Committee of the American College of Physicians (2015). Risk assessment and prevention of pressure ulcers: a clinical practice guideline from the American College of Physicians. Ann. Intern. Med..

[22] Fujiwara H., Isogai Z., Irisawa R., Otsuka M., Kadono T., Koga M. (2018). Wound, Pressure Ulcer and Burn Guidelines - 2: Guidelines for the Diagnosis and Treatment of Pressure Ulcers.

